# Circulating Tumor Cell cluster phenotype allows monitoring response to treatment and predicts survival

**DOI:** 10.1038/s41598-019-44404-y

**Published:** 2019-05-28

**Authors:** Ajay Balakrishnan, Deepak Koppaka, Abhishek Anand, Barnali Deb, Gianluca Grenci, Virgile Viasnoff, Erik W. Thompson, Harsha Gowda, Ramray Bhat, Annapoorni Rangarajan, Jean Paul Thiery, K. Govind Babu, Prashant Kumar

**Affiliations:** 1 Institute of Bioinformatics, International Technology Park, Bangalore, 560066 India; 2 Manipal Academy of Higher Education (MAHE), Manipal 576104, Karnataka India; 3 Department of Medical Oncology, Kidwai Memorial Institute of Oncology, Bengaluru, India; 4 Mechanobiology Institute, National University of Singapore, Singapore, 117411 Singapore; 5 Department of Biological Sciences, National University of Singapore, Singapore, 117411 Singapore; 6 CNRS UMI3639, Singapore, 117411 Singapore; 7 School of Biomedical Sciences, Queensland University of Technology (QUT), Brisbane, Australia; 8 Institute of Health and Biomedical Innovation (IHBI) and Invasion and Metastasis Unit, Translational Research Institute (TRI), Queensland University of Technolgy, Woolloongabba, QLD 4102 Australia; 9 Department of Molecular Reproduction, Development and Genetics, Indian Institute of Science, Bengaluru, India; 10 Department of Biochemistry, Yong Loo Lin School of Medicine, National University of Singapore, Singapore, Singapore; 11 Comprehensive Cancer Center, Institut Gustave Roussy, 114 Rue Edouard Vaillant, Villejuif, France; 12 CNRS UMR 7057, Matter and Complex Systems, Université Paris Diderot, 10 rue Alice Domon et Léonie Duquet Paris, Paris, France

**Keywords:** Cancer screening, Metastasis

## Abstract

Circulating tumor cells (CTCs) are putative markers of tumor prognosis and may serve to evaluate patient’s response to chemotherapy. CTCs are often detected as single cells but infrequently as clusters and are indicative of worse prognosis. In this study, we developed a short-term culture of nucleated blood cells which was applied to blood samples from breast, lung, esophageal and bladder cancer patients. Clusters of different degrees of compactness, classified as very tight, tight and loose were observed across various cancer types. These clusters show variable expression of cytokeratins. Cluster formation from blood samples obtained during the course of chemotherapy was found to be associated with disease progression and shorter overall survival. The short-term cultures offer a robust and highly reliable method for early prediction of treatment response in different cancer types.

## Introduction

Metastasis contributes to 90% of cancer deaths^1^. Circulating tumor cells (CTCs) are cancer cells disseminated in blood and are considered to be pivotal in the metastatic cascade^2^. Studies on CTCs have gained momentum since last few years. However, a deeper understanding about the biological and clinical impact of CTCs in the metastatic process is still awaiting. Isolation of pure CTC population is still a major challenge as they represent only a minor fraction of white blood cells. Enrichment methods based on physical and biological properties of CTCs have not resolved the issue of heterogeneity in size, deformability and surface marker expression among other parameters. In particular, it has been difficult to estimate the number of CTCs expected to be released in each cancer type^3,4^. Inter and intra-tumor heterogeneity introduces significant challenges to characterize these rare populations of cancer cells^5^. Recent studies have documented that CTC clusters, although infrequently detected, could promote their longer-term survival in the blood stream and increase their metastatic potential^6–14^. For instance, it has been shown that CTC clusters have 50 times increased metastatic potential than a single CTC^12^. CTC clusters have been shown to engage in epithelial-mesenchymal transition (EMT) through large amounts of TGF beta released by platelets co-aggregated in these clusters^15^. The acquisition of an EMT status in the CTC clusters may augment chemoresistance^16^. In addition, platelets will contribute to the formation of a favourable metastatic microenvironment through the recruitment of granulocytes in CTC microemboli^17^. Another study reported the presence of giant macrophages associated with CTC possibly promoting their dissemination and survival in the circulation, together supporting the significance of the microenvironment in tumor growth, progression and metastasis^18^. In our previous study, we observed that in short-term cultures CTCs could form clusters comprising other cell types including macrophages and natural killer-like cells^19^.

We recently established a short-term culture method using laser-ablated microwells that permits the expansion of CTCs from whole blood of breast cancer patients^19^. CTCs that were able to survive in short-term culture were shown to form multilayered cell clusters in association with NK cells and macrophages, possibly reflecting some association *in vivo* as described above. In this study, we used similar microwells, molded in agar to culture erythrocyte-lysed whole blood preparations from cancer patients having locally advanced or metastatic lung, breast, esophagus, bladder and hemangioendothelioma. We have further developed a robust method to quantify the CTC cluster formed across cancer types and during various treatment regimens. These clusters were categorized into three cluster types based on the cell density. We utilized the cluster phenotype as a predictive indicator of treatment response and to determine the therapeutic efficacy of the drugs.

## Results

### CTC culture in agar microwells

**Figure 1:**
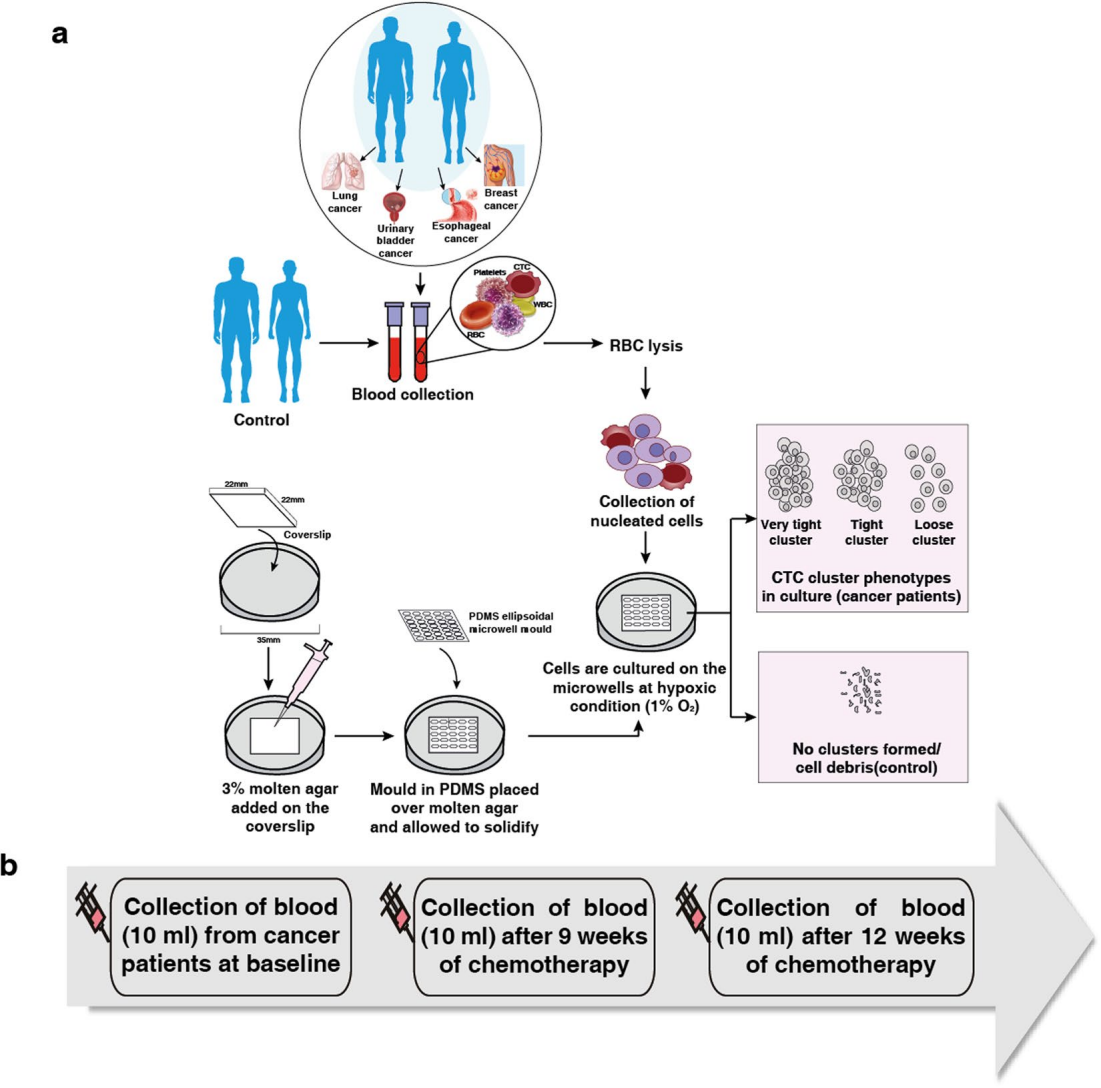
Workflow (**a**) Methodology for the culture of nucleated cells from blood in microwells. Blood samples from lung, bladder, esophageal and breast cancer patients were depleted of RBC before plating into dishes containing a coverslip coated with agar elipsoidal microwell designed using a PDMS mold. Nucleated cells were cultured for 21 days under hypoxic (1% oxygen) and scored/monitored for cluster formation (**b**) Time-line for collection of blood at three time-points from lung and breast cancer patients.

**Figure 2:**
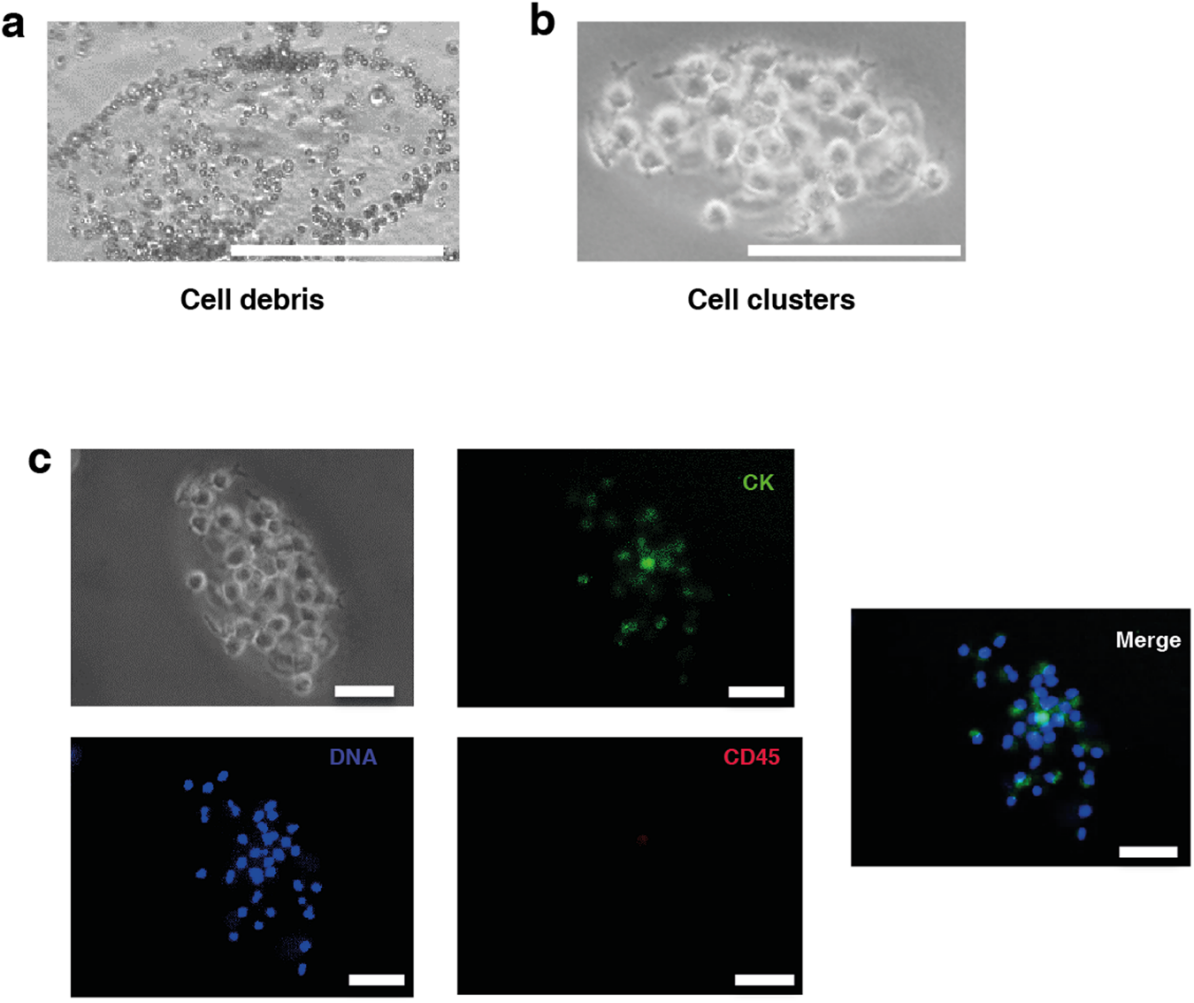
(**a**) Cell debris derived from dead white blood cells. (**b**) Formation of CTC clusters from blood of lung cancer patient (Scale bar: 200 µm). (**c**) Immunostaining of CTCs cultured from lung cancer patient for pan-cytokeratin (CK) and CD45; DAPI was used to counterstain nuclei. (Scale bar: 100 µm).

In order to evaluate cell cluster formation, the nucleated cell fraction after RBC lysis was cultured from healthy and cancer patient (lung, breast, bladder and esophageal cancers) blood samples for 21 days as shown in (Fig. 1a). At day 14, we observed cell debris generated by non-proliferative cultures (Fig. 2a) or multilayered, clusters (Fig. 2b). These clusters showed variable cytokeratin (CK) staining. CK + ve cells were negative for CD45 and there was a clear enrichment of CK^+^/CD^45−^ cells after day 14 cultures (Fig. 2c).

### CTC cluster formation and characterization

**Figure 3:**
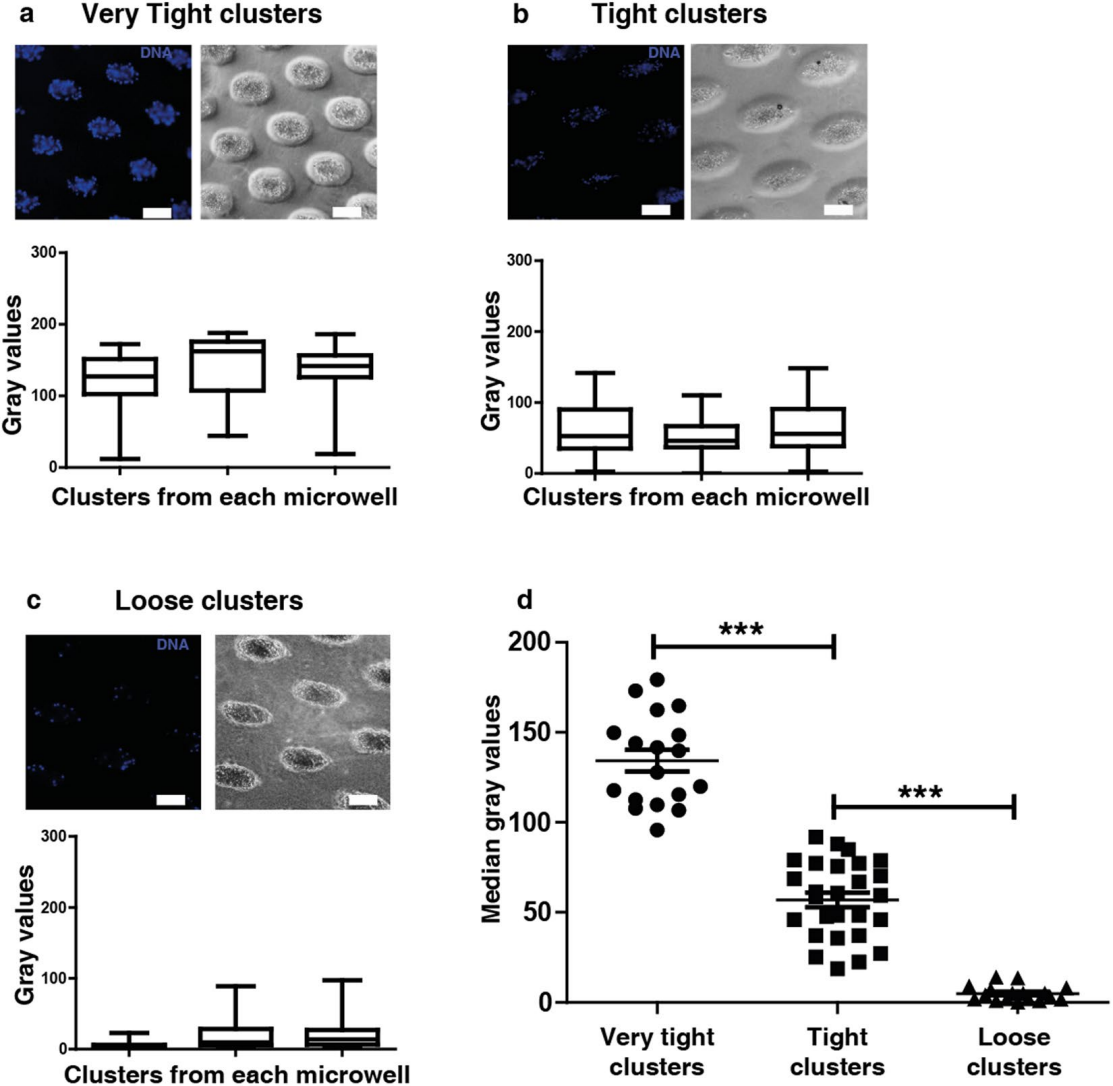
Classification of clusters formed in microwells. (**a**) Very tight clusters (**b**) tight clusters (**c**) loose clusters based on cell density using fluorescent intensity of DAPI (gray value). (**d**) Median gray values of clusters formed from each well of different cluster types (Very tight cluster n = 18 clusters; tight cluster n = 27 clusters; loose cluster n = 17 clusters). Data are presented as mean ± SEM (***p < 0.001) (Scale bar: 200 µm).

We next examined whether distinct cluster phenotypes appear in culture of blood samples from patients bearing different cancer types. We classified these clusters on the basis of their density or compactness using the fluorescence intensity values of DAPI (gray value). The clusters were categorized based on their compactness into 3 different types: very tight, tight and loose (Fig. 3a–c). The statistical significance for each cluster type is shown in (Fig. 3d). We observed predominantly tight clusters from breast and lung cancer patients; on the other hand, loose CTC clusters formed with blood samples from esophageal and bladder cancer patients (Supplementary Figs S1 and S2, Supplementary Table S1).

### Correlation of cluster formation with treatment response and efficacy

Cluster formed by patient blood samples were investigated for its correlation with drug response. To further correlate the cluster formation and treatment response, serial (three samples were collected from 17 breast and 12 lung cancer patients) on 9^th^ and 12^th^ week of therapy (Fig. 1b). This cohort comprised metastatic breast and lung cancer patients that were treated with various drug regimens (Supplementary Table S1). We observed a progressive reduction in cluster formation from patient samples, correlating with treatment response.

**Figure 4:**
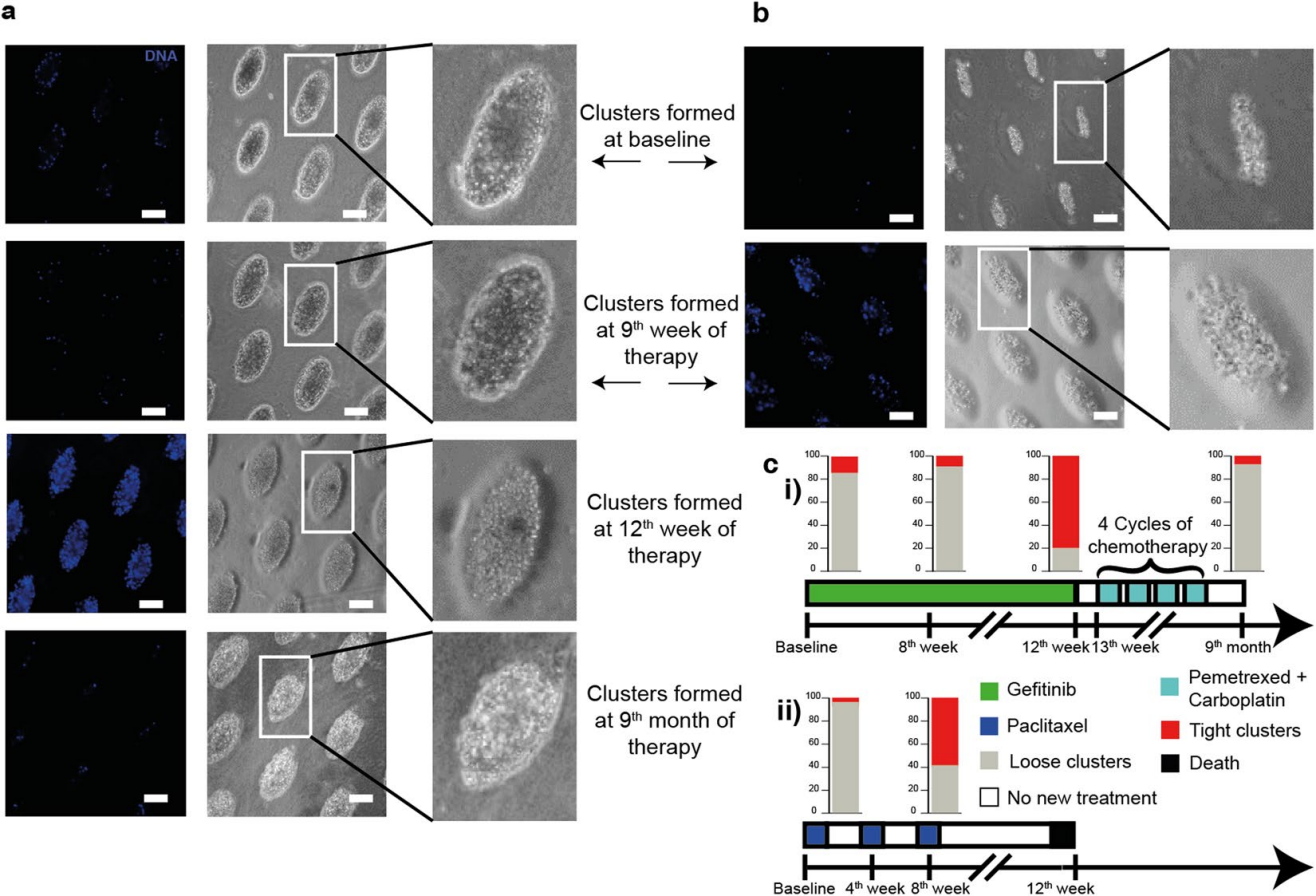
Cluster formation (day 14) in cancer blood sample at various time-points during follow up with treatment schedule for (**a**) lung cancer patient (**b**) breast cancer patient and (**c**) the schematic of treatment schedule for (i) lung and (ii) breast cancer patient.

**Figure 5:**
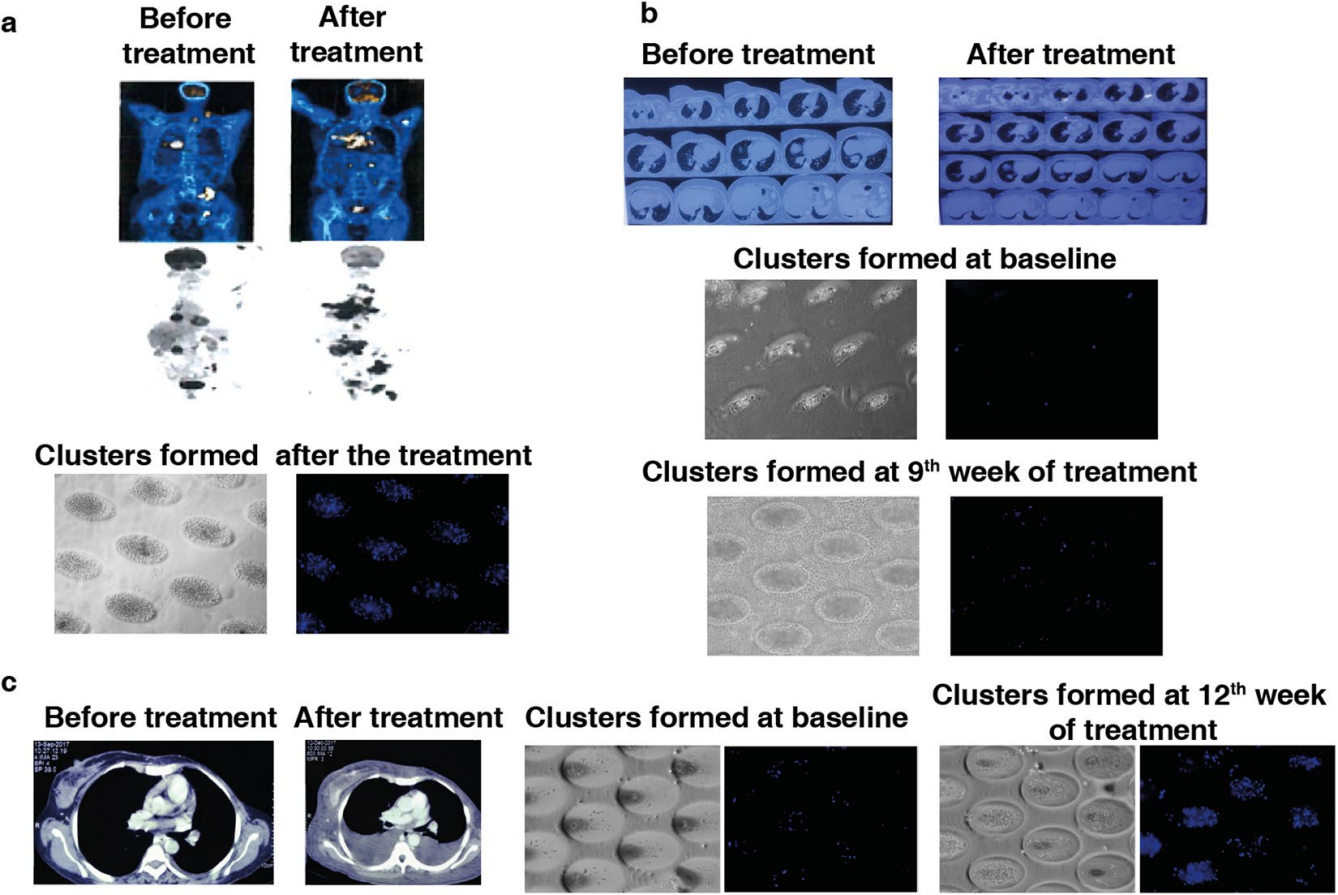
Clinical correlation of cluster formation in (**a**) lung cancer patient developing hyper progression (by PET/CT scan) on nivolumab treatment for 4 cycles (**b**) breast cancer patient with no sign of cancer progression (by PET scan) before and after treatment for 2 cycles (**c**) breast cancer patient with progressive cancer (by CT scan) before and after treatment for 4 cycles.

We also analyzed blood samples from unique cases with lung and breast cancers. The blood sample collected from a lung cancer patient at baseline as well as at the 9^th^ week of gefitinib treatment (targeting mutated EGFR), produced loose clusters, however, we observed tight clusters on the 12^th^ week of the therapy. Interestingly, a blood sample from the same patient on the follow-up after 9^th^ month of the treatment produced loose clusters in culture. Our observation suggested that the patient responded to gefitinib (Fig. 4a,c(i)). In contrast, while a breast cancer patient sample gave rise to loose clusters at baseline, tight clusters were formed from a blood sample taken during the mid-chemotherapy cycle (gemcitabine + paclitaxel treatment) (Fig. 4b,c(ii)). This patient eventually did not survive. Another unique case concerned a lung cancer patient who progressed following nivolumab-based immunotherapy. The culture formed from this patient produced tight clusters (Fig. 5a), the patient was reassessed by PET and CT scans post 4 cycles of nivolumab and was reported with multiple metastasis. The patient died during the follow-up period. In another example, a breast cancer patient treated with gemcitabine and carboplatin did not present any progressive disease by PET scan (Fig. 5b). Loose clusters formed from blood taken during both pre and post chemotherapy treatments from this patient showed similar observation. Also, another sample from breast cancer patient with progressive disease as shown by CT scan, formed tight cluster after chemotherapy (paclitaxel treatment) and the patient did not survive (Fig. 5c). Thus, cluster formation could also be a predictor of response to different therapeutic regimens in cancer patients.

### Correlation of patient survival with cluster formation

**Figure 6:**
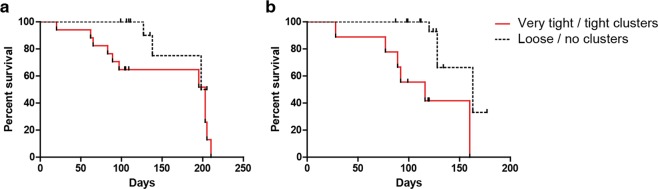
Comparison of survival in (**a**) Lung and (**b**) Breast cancer patients with and without cluster formation.

To correlate cluster formation with survival, samples were obtained from 34 patients with breast cancer and 31 patients with lung cancer. These patients were enrolled, when they were presented with progressive disease since their last treatment regimen but before commencing a new treatment regimen. Blood was collected when the patients were treatment naive, 9^th^ week and 12^th^ week after treatment. It is interesting to note that tight cluster formation correlated with shorter patient survival. The Kaplan-Meier survival analysis showed that cluster formation in both advanced stage lung and breast cancer patients who have undergone treatment correlated with shorter overall survival (Fig. 6a, b).

## Discussion

Enumeration of CTCs can offer a simple, robust method for diagnosis, prognosis and monitoring of treatment response in advanced cancer patients^20–28^. In the last decade, several studies have described methods of isolation of these rare cell populations. However, CTCs are still contaminated by leukocytes and each method cannot capture all the putative CTCs. In fact, CTCs are often detected at very low levels in some cancers and it is noteworthy that a high proportion of blood samples from metastatic patients remain negative. Most of the pre-enrichment approaches result in the loss of CTCs thus so far failed to retrieve an adequate number of CTCs for further analysis. In a previous study from our group, putative CTCs were cultured without prior enrichment^19^. The method provided higher efficiency and accuracy to enrich CTCs through short-term cultures. Here, we have modified the culture method using agar rather than tapered microwells to produce the microwells more easily and to improve their follow-up by microscopy. We employed our culture method for *ex-vivo* expansion of CTCs from patients with breast, lung, esophagus and bladder cancer.

Our culture system enabled us to successfully expand CTCs without prior enrichment. The method requires 2.5 ml blood per 35-mm dish to expand CTCs. Microwells were easy to establish and replicate with minimal set-up in a laboratory. We maintained the culture under hypoxic conditions. We used the proliferative potential of the cultured cells in these microwells to access the cluster phenotype in longitudinal post-treatment samples of breast and lung cancer patients. These patients had undergone subsequent chemotherapy and the cluster phenotype may reflect the treatment efficacy. We observed *in vitro* expansion of CTCs from patients with early stage, locally advanced, or metastatic cancer conditions. Interestingly, we noticed three different phenotypes of these clusters based on the cluster density: very tight, tight and loose. These clusters demonstrated a phenotype that was CK + ve/CD45 − ve. We found loose clusters from the blood of patients who had no clinically measurable disease while tight or very tight clusters were formed in blood of the patients with advanced disease. A number of cells in these clusters expressed CK but were not positive for CD45.

This cluster phenotype could be used to guide and select a specific drug for effective treatment for patient. Interestingly, we observed different cluster phenotypes formed in microwells across these four cancer types. The loose clusters in the microwells from post-treatment samples may suggest that the treatment efficacy is optimal and may probably reflect low number of cancer-initiating cells. On the other hand, the tight or very tight clusters suggest that there could be a higher number of cancer-initiating cells refractory to treatment. However, we did not notice significant difference in cluster diameter from breast and lung cancer blood samples each forming very tight clusters. (Supplementary Fig. S3).

We next aimed to link clinicopathological features with cluster formation. We observed that a favourable therapy resulted in loose cluster formation while the tight cluster suggested therapy resistance. In addition, these clusters show different phenotypes across 4 cancer types that could be correlated with the number of CTCs reported in each cancer type^20,29,30^. We have enumerated CTCs using pan-CK positive, CD45 negative and DAPI-positive cells in clusters observed on day14 (Supplementary Table S2). The CTCs enumerated from lung and breast cancer patients, showed a similar range with the previously published studies. Nevertheless, we understand the limitation of the enumeration based on an epithelial marker. In our previous study, we had observed that in some cases CK positive staining was not observed in the initial clusters but we could see them in day 14 clusters^19^. In breast and lung cancer patient samples (Supplementary Table S3), we observed very tight and tight clusters showing a clear correlation with poor patient survival. The culture from advanced stage breast cancer patient who did not survive exhibited a tight cluster (Supplementary Fig. 4a). In a recent study by Muralidar *et al*., reported a similar finding where the clusters were predictive of poor prognosis in early stage of lung cancer patients^31^. We also obtained one of the samples from a patient having malignant epithelioid hemangioendothelioma, a rare tumor of vascular origin. The culture gave raise to tight clusters and the patient deceased during follow-up (Supplementary Fig. 4b). To the best of our knowledge, this study is one of the few, which suggests the possible role of cluster formation in patient survival. The short-term culture method described in this study was very effective in assessing patients with different cancer types, treatment choice and metastatic potential.

In conclusion, dynamic changes of the CTC cluster phenotype in culture makes it a relatively straightforward approach to monitor an association with cancer progression and patient survival. It will provide a proxy to study drug responses *in vitro* and will boost the clinical utility of liquid biopsy^32–39^. Although a larger sample cohort must be studied for each of the cancers analyzed in this study, it represents a leap-forward in correlating the phenotypic characteristics of CTC clusters in culture with the patients’ status. We believe that the method is exploitable and could be used as a prognostic and predictive tool in identifying therapy response or resistance.

## Materials and Methods

### Sample collection

Blood samples were obtained from 86 breast cancer patients, 52 lung cancer patients, 6 esophageal cancer patients, 6 bladder cancer patients and 1 hemangioendothelioma cancer patient. This study was approved by our Medical Ethics Committee (MEC; institutional review board and local ethics committee), Kidwai Memorial Institute of Oncology (KMIO/MEC/017/23.March.2017, KMIO/MEC/018/23.March.2017, KMIO/MEC/011. November.2016) and confirmed that all research was performed in accordance with relevant guidelines and regulations. Informed consent was obtained from all the participants and/or their local guardian for inclusion in this study. All the blood samples were collected in sterile EDTA-coated vacutainer tubes (BD Vacutainer®) and kept on ice. The clinical treatment characteristics for the four cohorts and unique cases are summarized in Supplementary Table S1.

### Sample processing

Fresh blood was collected in EDTA coated vacutainer tubes (BD Vacutainer®) and processed within 5 hrs of the blood collection to minimize blood clotting. The whole blood was separated by centrifugation and the remaining cell fraction was subjected to RBC lysis by RBC lysis buffer (155 mM NH_4_Cl + 10 mM KHCO_3_ + 127 μM EDTA) with gentle mixing for 10 mins. The blood was suspended in RBC lysis buffer in 1:2 ratio. It was then centrifuged to remove RBC fragments and washed once with sterile phosphate-buffered saline (PBS). Nucleated cells were re-suspended in Dulbecco’s modified Eagle medium (DMEM) (Sigma) supplemented with 10% fetal bovine serum (FBS) (Life Technologies) and 1% penicillin-streptomycin (Sigma).

### Preparation of the ellipsoidal molds and culture assays

A general strategy for the culture of nucleated cells from patient samples in ellipsoidal microwells is shown in (Fig. 1). The microwells were prepared by placing a cover slip (22 mm × 22 mm) on a 35 mm culture dish. Molten agar (3%) was poured on the top of the cover slip enough to cover it (500 µl approx). The ellipsoidal microwell mold in PDMS (polydimethylsiloxane) was then placed on the top of the cover slip containing the molten agar and allowed to solidify. The culture conditions were grown at 37 °C, 5% CO_2_ in humidified (Galaxy^®^ 48R, Eppendorf) (1% O_2_). The culture medium was replaced every 48–72 hrs with minimal disturbances to the microwells. Cultures were maintained for 3 weeks and imaged on day 7, 14, 21 with phase contrast microscope (Olympus IX71) and analyzed the images using ImageJ (NIH, Bethesda, MD).

### Characterization of CTC Clusters

The samples were maintained at 37 °C in 5% (V/V) CO_2_ and 1% O_2_ hypoxia (Galaxy^®^ 48R, Eppendorf) under humidified conditions. The samples were cultured in high-glucose Dulbecco’s modified Eagle’s medium (DMEM) supplemented with 10% fetal bovine serum (FBS) and 1% penicillin-streptomycin (all from Invitrogen, Carlsbad, CA). Each processed sample (10 ml of whole blood) was split and seeded in three 35 mm dish containing the microwells.

The threshold values to classify the clusters were calculated from the median of grey values from each cluster in a microwell, which reflects the fluorescent intensity of DAPI in clusters. In majority of cases from lung and breast CTC clusters, the median grey score obtained were more than 100, which were categorized as very tight clusters, followed by grey score ranging between 10–100 as tight clusters. In the case of esophagus and bladder CTC clusters, the median grey score was below 10 and was classified as loose clusters (Supplementary Fig. S5).

### Immunophenotyping of CTC clusters

Cells/clusters grown in the agar mold were fixed with 4% paraformaldehyde (PFA) (Merk, India) for 20 mins and permeabilized with 0.1% Triton X-100 (Himedia, India) in PBS. The cells were then treated by blocking buffer (3% bovine serum albumin (BSA) in PBS) for 1 hour. Cells were incubated with CD45 (368502; BioLegend, USA), Pan-CK (ab9377; Abcam) primary antibodies overnight at 4 °C and then incubated with anti-mouse secondary antibody conjugated with Alexa 568 (Invitrogen, USA) and anti-rabbit secondary antibody conjugated with Alexa 488 (Invitrogen, USA). Cells were then counter-stained with DAPI (4′,6-Diamidine-2′-phenylindole dihydrochloride) (Invitrogen, USA) and the epifluorescent images were acquired under Olympus IX71 microscope. The images were analyzed by ImageJ [National Institutes of Health (NIH)]. The clusters were classified according to the median gray values. The median values were tested for statistical significance by 2-tailed t-test.

## Supplementary information

Supplementary Dataset 1
