# Supplementary material for: Circulating Tumor Cell cluster phenotype allows monitoring response to treatment and predicts survival
Source: Sci Rep. 2019 May 28;9:7933. doi: 10.1038/s41598-019-44404-y (PMC6538674; doi:10.1038/s41598-019-44404-y)
Supplement: Supplementary file 1 — Supplementary Dataset 1 [file 41598_2019_44404_MOESM1_ESM.pdf]

**Circulating Tumor Cell cluster phenotype allows monitoring response to treatment and predicts survival**

Ajay Balakrishnan, Deepak Koppaka [Ɨ], Abhishek Anand [Ɨ], Barnali Deb, Gianluca Grenci, Virgile Viasnoff, Erik Thompson, Harsha Gowda, Ramray Bhat, Annapoorni Rangarajan, Jean Paul Thiery[*], Govind Babu K[*], Prashant Kumar[*]

[Ɨ] **Contributed equally**

***Corresponding Authors**

**Supplementary Fig. S1** Comparison of clusters formed from lung, breast, bladder and esophageal cancer patients

**Supplementary Fig. S2** Phase contrast microscopic images of clusters formed by lung, breast, bladder and esophageal cancer patients' blood samples (Scale bar: 200 µm)

**Supplementary Fig. S3** Comparison of cluster diameters from lung and breast cancer patients

**Supplementary Fig. S4** Clusters from **A)** breast cancer patient **B)** hemangioendothelioma patient who did not survive subsequently (Scale bar: 200 µm)

**Supplementary Fig. S5** Clusters formed from each patient in **A)** Lung **B)** Breast **C)** Bladder and **D)** Esophageal cancer

**Supplementary Table S1** List of drug administered to lung and breast cancer patient cohorts included in the study

**Supplementary Table S2** CTC enumeration from breast and lung cancer patients compared with previously published results.

**Supplementary Table S3** List of patients with the type of clusters formed in the microwell culture system

**Supplementary Figure S1**

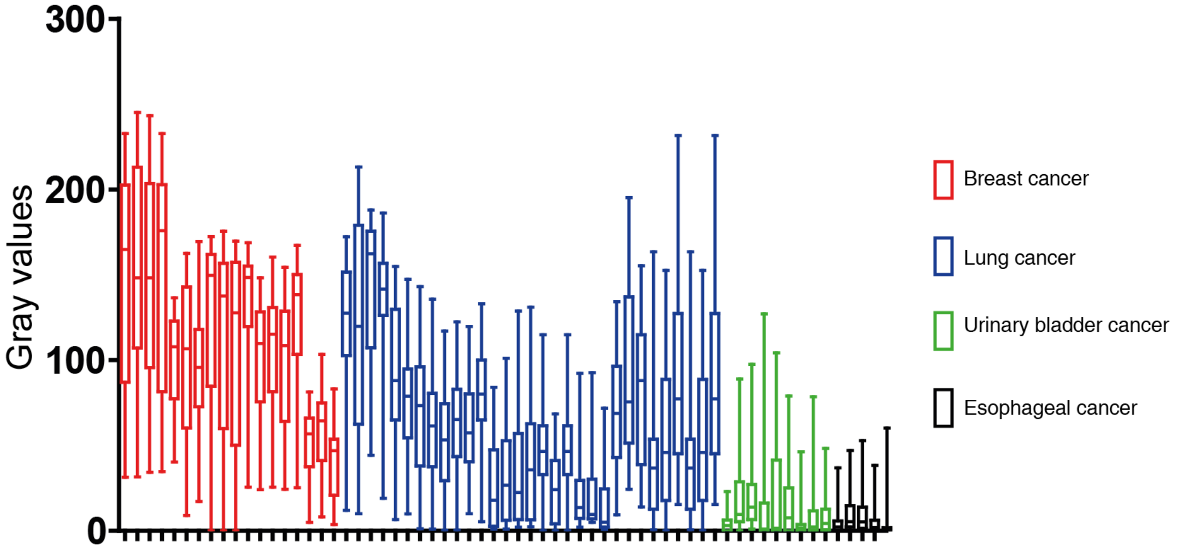

## Supplementary Figure S2

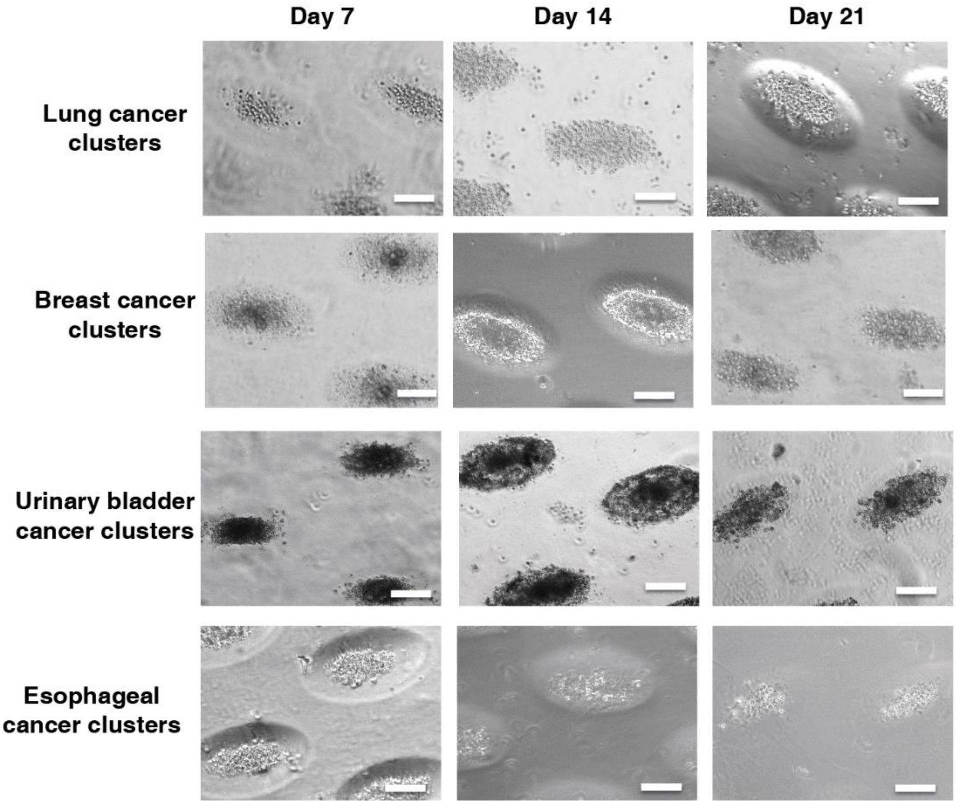

# Supplementary Figure S3

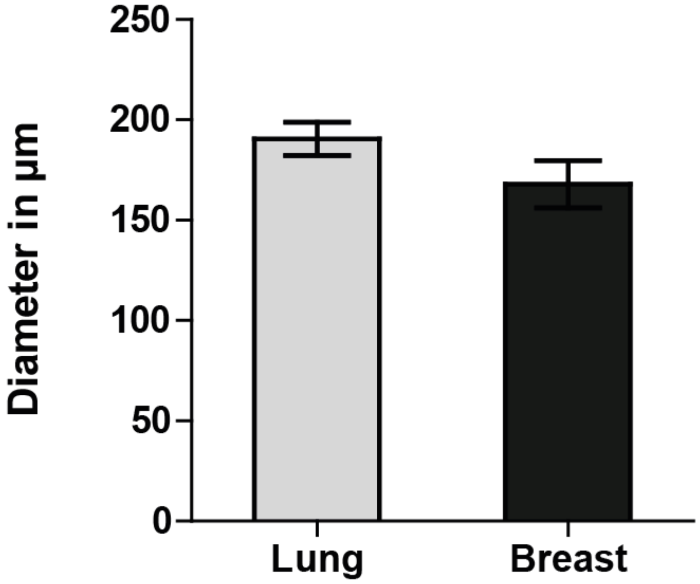

# Supplementary Figure S4

**a**

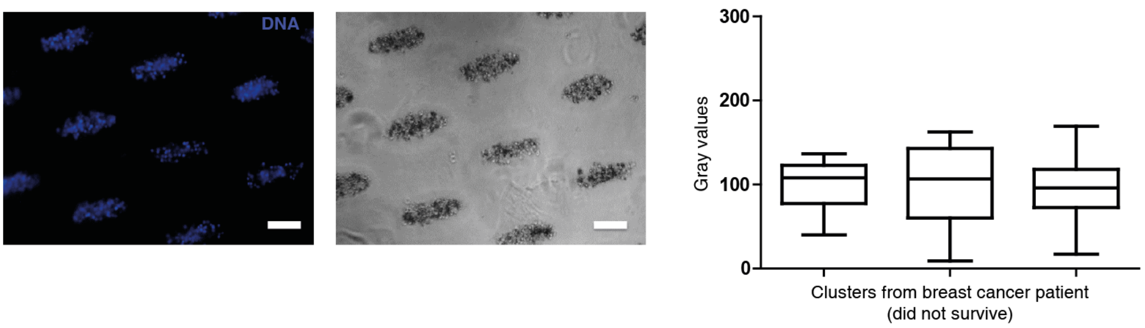


**b**

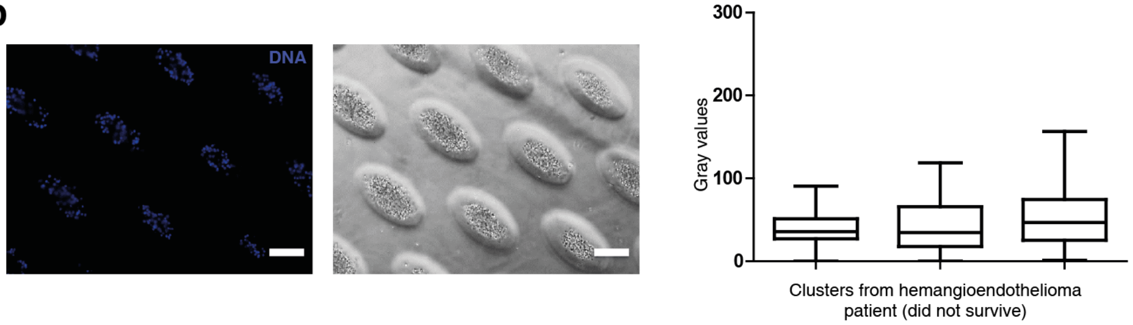

## Supplementary Figure S5

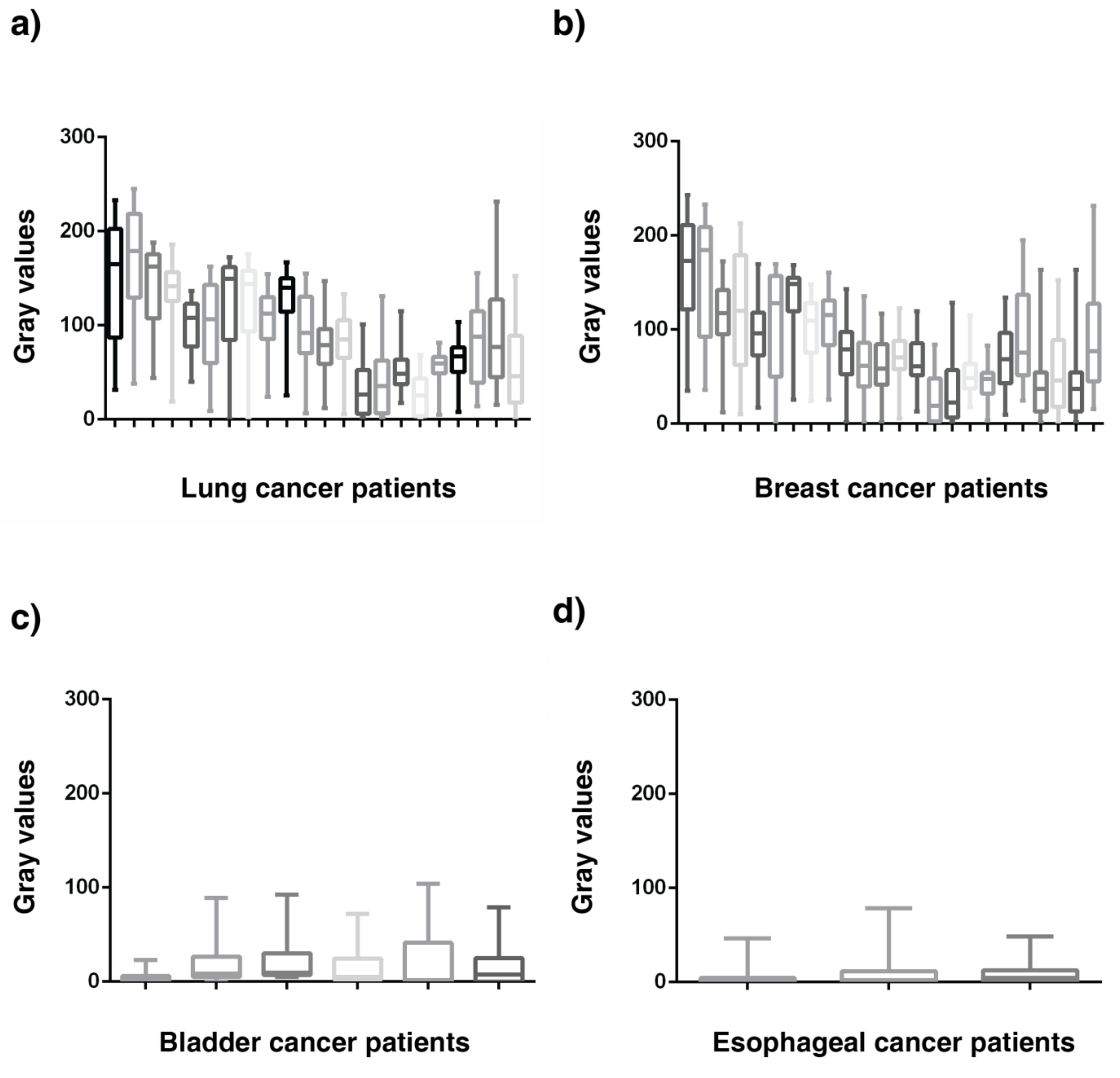

# Supplementary Table S1

| **Drugs used for breast cancer patient cohort (No. of patients)** | **Drugs used for lung cancer patient cohort (No. of patients)** |
|---|---|
| Paclitaxel (8) | Pemetrexed + Carboplatin (18) |
| Capecitabine (1) | Gefitinib (14) |
| Anastrazole (3) | Nab-paclitaxel + Carboplatin (2) |
| Gemcitabine + Carboplatin (4) | Gemcitabine+ Carboplatin (6) |
| Tamoxifen (4) | CDDP + etoposide (1) |
| Flurouracil + Epirubicin + Cyclophosphamide (10) | |
| Letrozole + Palbociclib (1) | |
| Docetaxel + Adriamycin + Cyclophosphamide (1) | |
| Cyclophosphamide + Methotrexate + Fluorouracil (1) | |
| Docetaxel + Trastuzumab (1) | |
| Letrozole (2) | |
| Docetaxel (4) | |

## Supplementary Table S2

| S .no. | Cancer type; no. of patients included in the study | Number of CTCs in the culture (per 10 ml of blood) on 14th day of culture | Number of CTCs reported in previous studies; no. of patients included in the study | Reference |
|---|---|---|---|---|
| 1 | Breast; 86 | 61-80 | 5-100 per 1 ml of blood; 10 | 36 |
| | | | 3-3000 per 6 ml of blood; 6 | 35 |
| | | | 1.0 - 202.7 per 1 ml of blood; 181 | 37 |
| | | | 0.75-23.25 per 1 ml of blood; 22 | 38 |
| 2 | Lung; 52 | 86-100 | 5-100 per 1 ml of blood; 55 | 36 |
| | | | 1-11 per 1 ml of blood; 19 | 39 |
| | | | 0.5-24.2 per 1 ml of blood; 15 | 38 |

**Supplementary Table S3**

| Pt No | Cancer type | Cluster type | Survival (Y/N) |
|---|---|---|---|
| 1 | Breast | Very Tight | |
| 2 | Lung | Very Tight | N |
| 3 | Breast | Very Tight | N |
| 4 | Breast | Very Tight | N |
| 5 | Breast | Very Tight | |
| 6 | Lung | Tight | |
| 7 | Lung | Tight | Y |
| 8 | Lung | Loose / no clusters | Y |
| 9 | Lung | Tight | N |
| 10 | Breast | Tight | |
| 11 | Lung | Tight | Y |
| 12 | Lung | Tight | Y |
| 13 | Lung | Tight | Y |
| 14 | Bladder | Loose / no clusters | |
| 15 | Lung | Loose / no clusters | |
| 16 | Bladder | Loose / no clusters | |
| 17 | Esophagus | Loose / no clusters | |
| 18 | Esophagus | Loose / no clusters | |
| 19 | Esophagus | Loose / no clusters | |
| 20 | Lung | Tight | N |
| 21 | Breast | Loose / no clusters | Y |
| 22 | Esophagus | Loose / no clusters | |
| 23 | Esophagus | Loose / no clusters | |
| 24 | Lung | Loose / no clusters | N |
| 25 | Lung | Loose / no clusters | Y |
| 26 | Breast | Loose / no clusters | N |
| 27 | Breast | Loose / no clusters | Y |
| 28 | Breast | Loose / no clusters | Y |
| 29 | Lung | Tight | Y |
| 30 | Lung | Loose / no clusters | Y |
| 31 | Bladder | Loose / no clusters | |
| 32 | Breast | Loose / no clusters | N |
| 33 | Breast | Loose / no clusters | Y |
| 34 | Breast | Loose / no clusters | Y |
| 35 | Lung | Tight | Y |
| 36 | Breast | Loose / no clusters | Y |
| 37 | Breast | Tight | Y |
| 38 | Breast | Tight | Y |
| 39 | Breast | Loose / no clusters | Y |
| 40 | Breast | Loose / no clusters | Y |
| 41 | Breast | Loose / no clusters | Y |
| 42 | Esophagus | Loose / no clusters | |
| 43 | Breast | Loose / no clusters | Y |
| 44 | Lung | Tight | N |
| 45 | Lung | Loose / no clusters | N |
| 46 | Lung | Loose / no clusters | Y |

| | | | |
|---|---|---|---|
| 47 | Bladder | Loose / no clusters | |
| 48 | Breast | Loose / no clusters | Y |
| 49 | Breast | Tight | Y |
| 50 | Lung | Loose / no clusters | |
| 51 | Lung | Tight | N |
| 52 | Breast | Loose / no clusters | Y |
| 53 | Lung | Tight | N |
| 54 | Lung | Tight | N |
| 55 | Lung | Tight | N |
| 56 | Lung | Tight | N |
| 57 | Lung | Tight | N |
| 58 | Lung | Tight | N |
| 59 | Breast | Tight | N |
| 60 | Breast | Tight | N |
| 61 | Breast | Tight | N |
| 62 | Breast | Tight | N |
| 63 | Lung | Loose / no clusters | N |
| 64 | Breast | Loose / no clusters | N |
| 65 | Breast | Loose / no clusters | N |
| 66 | Lung | Loose / no clusters | Y |
| 67 | Lung | Loose / no clusters | Y |
| 68 | Lung | Loose / no clusters | Y |
| 69 | Lung | Loose / no clusters | Y |
| 70 | Lung | Loose / no clusters | Y |
| 71 | Lung | Loose / no clusters | Y |
| 72 | Breast | Loose / no clusters | Y |
| 73 | Breast | Loose / no clusters | Y |
| 74 | Breast | Loose / no clusters | Y |
| 75 | Breast | Loose / no clusters | Y |
| 76 | Breast | Loose / no clusters | Y |
| 77 | Breast | Loose / no clusters | Y |
| 78 | Breast | Loose / no clusters | Y |
| 79 | Breast | Loose / no clusters | Y |
| 80 | Breast | Loose / no clusters | Y |
| 81 | Breast | Loose / no clusters | Y |
| 82 | Breast | Loose / no clusters | Y |
| 83 | Bladder | Loose / no clusters | |
| 84 | Bladder | Loose / no clusters | |
| 85 | Breast | Loose / no clusters | |
| 86 | Breast | Loose / no clusters | |
| 87 | Breast | Loose / no clusters | |
| 88 | Breast | Loose / no clusters | |
| 89 | Breast | Loose / no clusters | |
| 90 | Breast | Loose / no clusters | |
| 91 | Breast | Loose / no clusters | |
| 92 | Breast | Loose / no clusters | |
| 93 | Breast | Loose / no clusters | |
| 94 | Breast | Loose / no clusters | |

| | | | |
|---|---|---|---|
| 95 | Breast | Loose / no clusters | |
| 96 | Breast | Loose / no clusters | |
| 97 | Breast | Loose / no clusters | |
| 98 | Breast | Loose / no clusters | |
| 99 | Breast | Loose / no clusters | |
| 100 | Breast | Loose / no clusters | |
| 101 | Breast | Loose / no clusters | |
| 102 | Breast | Loose / no clusters | |
| 103 | Breast | Loose / no clusters | |
| 104 | Breast | Loose / no clusters | |
| 105 | Breast | Loose / no clusters | |
| 106 | Breast | Loose / no clusters | |
| 107 | Breast | Loose / no clusters | |
| 108 | Breast | Loose / no clusters | |
| 109 | Breast | Loose / no clusters | |
| 110 | Breast | Loose / no clusters | |
| 111 | Breast | Loose / no clusters | |
| 112 | Breast | Loose / no clusters | |
| 113 | Breast | Loose / no clusters | |
| 114 | Breast | Loose / no clusters | |
| 115 | Breast | Loose / no clusters | |
| 116 | Breast | Loose / no clusters | |
| 117 | Breast | Loose / no clusters | |
| 118 | Breast | Loose / no clusters | |
| 119 | Breast | Loose / no clusters | |
| 120 | Breast | Loose / no clusters | |
| 121 | Breast | Loose / no clusters | |
| 122 | Breast | Loose / no clusters | |
| 123 | Breast | Loose / no clusters | |
| 124 | Breast | Loose / no clusters | |
| 125 | Breast | Loose / no clusters | |
| 126 | Breast | Loose / no clusters | |
| 127 | Breast | Loose / no clusters | |
| 128 | Breast | Loose / no clusters | |
| 129 | Breast | Loose / no clusters | |
| 130 | Breast | Loose / no clusters | |
| 131 | Breast | Loose / no clusters | |
| 132 | Lung | Loose / no clusters | |
| 133 | Lung | Loose / no clusters | |
| 134 | Lung | Loose / no clusters | |
| 135 | Lung | Loose / no clusters | |
| 136 | Lung | Loose / no clusters | |
| 137 | Lung | Loose / no clusters | |
| 138 | Lung | Loose / no clusters | |
| 139 | Lung | Loose / no clusters | |
| 140 | Lung | Loose / no clusters | |
| 141 | Lung | Loose / no clusters | |
| 142 | Lung | Loose / no clusters | |

| | | | |
|---|---|---|---|
| 143 | Lung | Loose / no clusters | |
| 144 | Lung | Loose / no clusters | |
| 145 | Lung | Loose / no clusters | |
| 146 | Lung | Loose / no clusters | |
| 147 | Lung | Loose / no clusters | |
| 148 | Lung | Loose / no clusters | |
| 149 | Lung | Loose / no clusters | |
| 150 | Lung | Loose / no clusters | |
| 151 | Hemangioendothelioma | Tight | N |
